# Facies architecture related to sea-level changes of the Upper Cretaceous (Santonian) Taneichi Formation, northeast Japan: evidence of actual sea-level fluctuation during the Late Cretaceous

**Published:** 2004-05-01

**Authors:** Koji Yagishita

**Affiliations:** Faculty of Education, Iwate University, 3-18-8, Ueda, Morioka, Iwate 020-8550

**Keywords:** Ravinement, regressive surface of erosion (RSE), ripples, the Santonian Taneichi Formation, incised valley system, herringbone (bipolar) cross-bedding

## Abstract

Well-exposed transgressive and regressive sequence of the Santonian Taneichi Formation occurs along the Pacific Coast of northeastern Honshu, Japan. Although the studied sequence is of only 60 m thickness, the outcrops show various sedimentary facies accompanied with two disconformities, suggesting a small-scale but substantial sea-level rise and fall. Orientations of some sedimentary structures, such as ripples and bipolar cross-stratification, indicate that the paleostrandline of the Taneichi Formation made a high angle with the present coastline. Compared with the Santonian Kunitan Formation that is exposed about 50 km south of the studied section and that consists of thick deposits due to an incised valley sedimentation, the thin sediments of the Taneichi Formation are thought to have been supplied from the broad coastal plain along the eastern margin of the Eurasian continent.

## Introduction

During the past two decades sequence stratigraphy has evolved into one of the most important concerns among sedimentologists. Recognition of the package of large-scale depositional units or systems tracts that are intimately related to the eustatic fluctuations has become a crucial tool for subsurface geology and petroleum exploration.1)

Another important aspects of the sequence stratigraphy is that the study has stimulated the analysis of sedimentary architecture at the coastal zone. Many papers have been reported to document the intriguing stratigraphy and sedimentary facies of depositional systems of the coastal and shallow marine components, such as the transgressive surface of erosion (ravinement) related to sea-level rise and the regressive surface of erosion (RSE) produced by sea-level fall.2),3) And they were newly testified to confirm the character of disconformities within the coastal stratigraphic record.

The sediments of the Upper Cretaceous Taneichi Formation exposed along the northern Sanriku coast show the various nearshore sedimentary facies associated with the small-scale but substantial sea-level rise and fall. In this paper the writer describes and discusses the facies analysis and disconformities within the sequence, which are related to the sea-level fluctuation in the formation.

## Geological setting

**Fig. 1:**
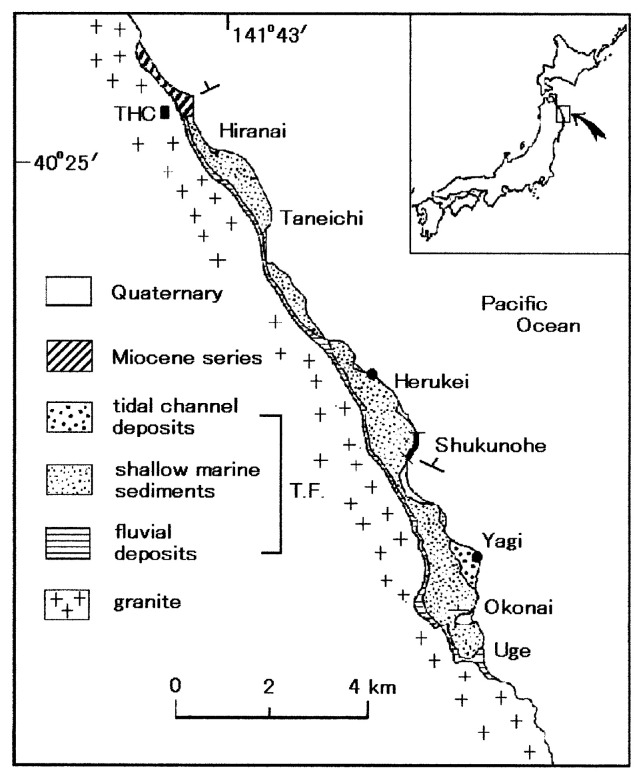
Distribution of the Taneichi Formation (fluvial, shallow marine and tidal channel deposits) along the Pacific coast. Closed circles denote the other outcrops of facies Spb except Shukunohe section (thick line). T.F.; Taneichi Formation, THC; Taneichi High School.

The Taneichi Formation is narrowly distributed along the northeastern edge of the Kitakami Massif (Mesozoic basement rocks), and the formation unconformably overlies the Early Cretaceous Hashigami Granite (Fig. 1). Although the northernmost part of the ‘formation’ is now assigned to belong to the Miocene,4) most sediments of the formation has long been dated as the Santonian. Compared with sediments formed in other Cretaceous forearc basins,5) total thickness of the formation is very thin, no more than 160 m. The main part of the formation consists of marine sediments which yield some fossils such as inoceramid (Bivalvia) species, *Sphenoceramus naumanni*, which was once erroneously called *S. sanrikuensis*,6) and *Paratexanites* (*Anatexanites*) sp. aff. *P*. (*A*.) *nomii* (Yabe et Shimizu).7) From palynological studies the very basal, non-marine part of the formation is dated as the Santonian, whereas the upper part of marine sediments may belong to the Early Campanian.8) Coastal bluffs of the Taneichi Formation at Shukunohe are well exposed, and the beds are only monoclinicly inclined with less than 15 ° dipping toward northeast (Fig. 1). With a few kilometers wave length, however, the formation of farther north and south of Shukunohe is gently folded, and such a tectonic structure allows us to see various sedimentary facies of the same stratigraphic level at different outcrops.

## Facies description and interpretation. Only

some representative facies codes and interpretations are documented herein. Other shallow marine sedimentary facies in the section, which include well-known hummocky cross-stratification,9) are not described in this paper.

### Facies Spb

**Fig. 2:**
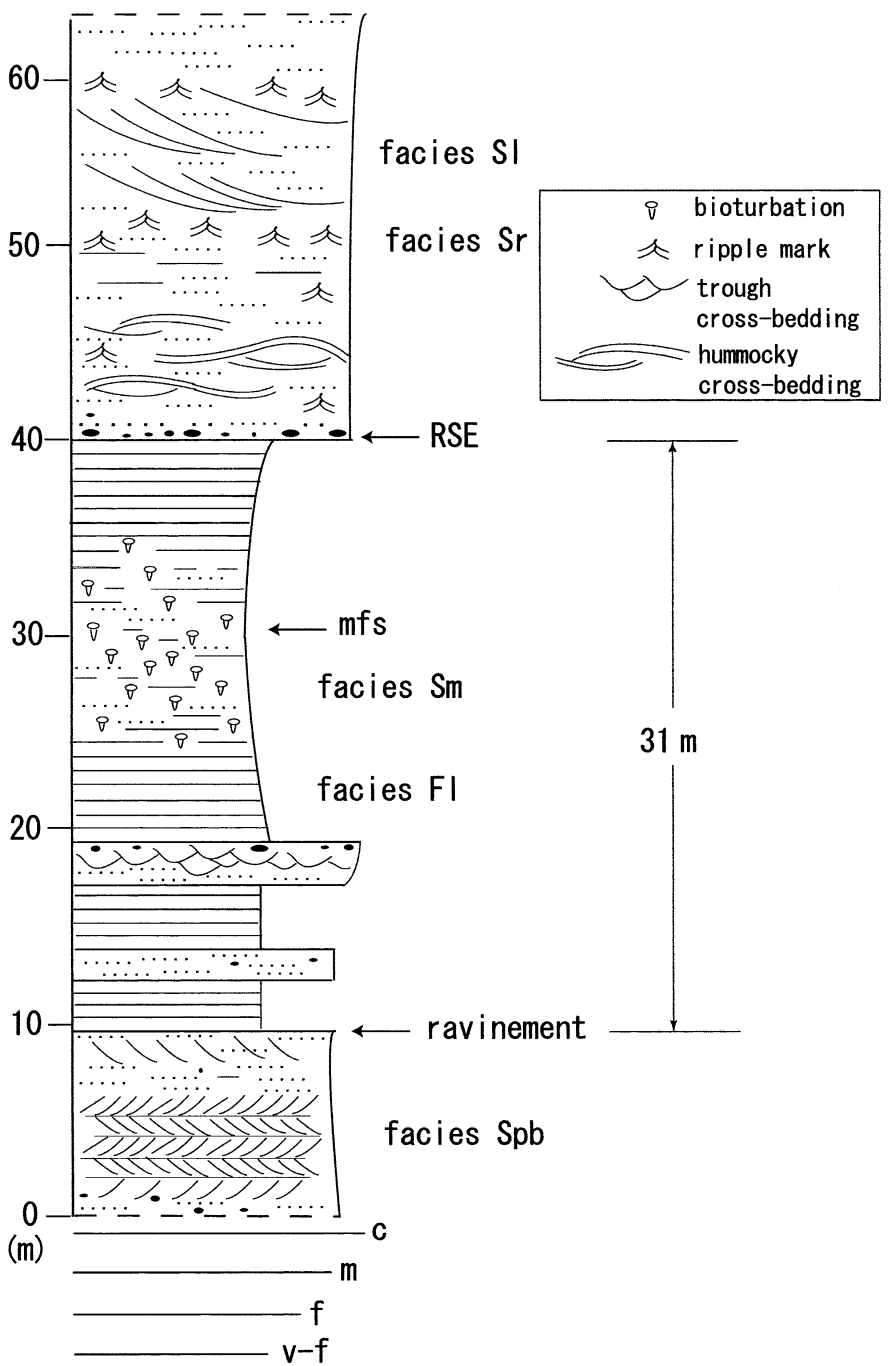
Columnar section of the Taneichi Formation at Shukunohe section. Thickness interval from ravinement to regressive surface of erosion (RSE) is less than a half of that observed at the Kunitan Formation, 50 km south of the studied section. mfs; maximum flooding surface.

**Fig. 3:**
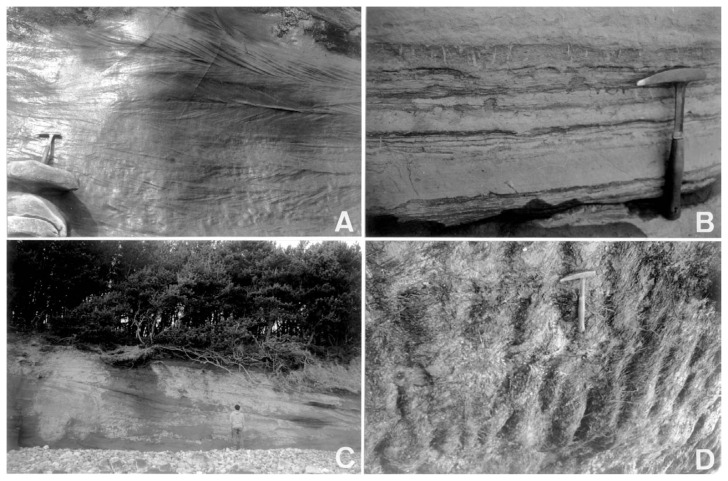
Representative sedimentary facies along Shukunohe section. The hammer in each outcrop is 0.3 m long. A. Facies Spb at the lowermost columnar section in Fig. 2. B. Bioturbated very fine-grained sandstone bed (facies Sm) and parallel laminated sandstone bed (facies Fl) at the middle part of the section in Fig. 2. C. Internal cross-stratification within sandwaves (facies Sl) at the upper part of the section in Fig. 2. Strike of the cross-bedding shows almost E-W orientation. D. Ripples (facies Sr) just above the regressive surface of erosion (RSE).

Bidirectional planar cross-bedding sandstone beds occur at the basal part of the columnar section (Figs. 2 and 3A). The decimeter scale set of this facies Spb, in which the representative foreset dips are 71 °/28 ° and 245 °/11 ° respectively. The sandstones consist of medium-grained, well-sorted detrital frameworks of lithic arenite.10)

The facies of bipolar cross-bedding, generally called herringbone cross-set (HBCS), suggests reversing currents in the tidal inlet. The tidal inlets laterally migrate along shore and the migration produces a fining-upward succession. However, due to ravinement the entire succession is not discernible at the outcrop. The facies also crops out at other localities (Fig. 1).

### Facies Fl

Parallel laminated of fine-grained sandstone and siltstone beds frequently occur in the lower and middle part of the columnar section. In the middle section, in particular, the facies Fl alternates with the bioturbated sandstone beds of facies Sm (Fig. 3B).

Following the stormy weather, the parallel laminae were made by settling out of suspended fine-grained materials. The facies is often made in the middle shoreface sedimentary environments. The facies in the deeper sedimentary environment, however, is subject to biogenic reworking, changing into the facies Sm.

### Facies Sm

The massive, fine-grained sandstone bed with bioturbation (facies Sm) alternated with the fine laminated siltstone bed of facies Fl (Fig. 3B). The beds of facies Sm are concentrated above and below the maximum flooding surface (mfs in Fig. 2).

Abundant trace fossils are well-preserved in the bioturbated beds, representing dwelling and locomotive activities of the fossils. One of such trace fossils is *Schaubcylindrichnus* ichnosp. (Narayama, personal communication).

### Facies Sl

The bedform is characterized with large-scale, low angle cross-stratification within the medium-grained sand-sized sediments (Figs. 2 and 3C). The basal part of cross-bedding is represented by a tangential contact with the substratum (Fig. 3C). The facies Sl occurs at the uppermost part of the columnar section (Fig. 2).

This type of bedform is called linear sand ridges or large-scale sandwaves, and the internal sedimentary structures, such as large-scale, low-angle cross-bedding, are generally discernible within the bedform.11) The internal cross-bedding planes dip seaward in the direction of progradation.

### Facies Sr

**Fig. 4:**
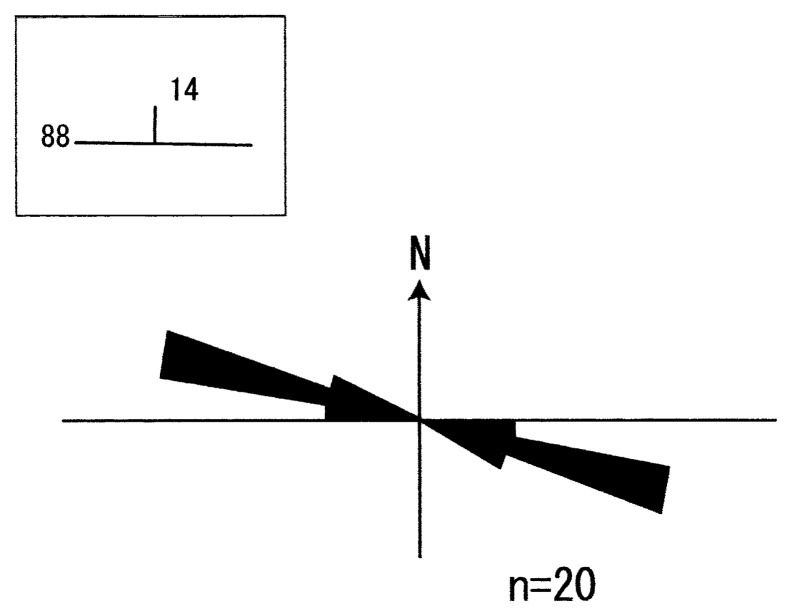
Circular histogram with 10° interval for ripple orientation at the uppermost section in Fig. 2. Strike and dip (N88°W14°N) in inset denote the cross-bedding orientation within sandwaves (facies Sl in Fig. 3C).

Symmetric ripples occur just above the regressive surface of erosion (RSE) and farther upward section of facies Sl (Fig. 2). Amplitude of the ripples is generally less than 0.05 m (Fig. 3D), and the ripples consists of medium- to coarse-grained sand. The average trend of ripple orientation is N61 °W, just above the regressive surface of erosion (RSE, based on 25 ripple measurements), whereas the trend is N75 °W at the uppermost section of Fig. 2 (20 measurements, Fig. 4).

Co-existence of facies Sr with facies Sl or facies Sr with hummocky cross-stratification suggests that facies Sr were made either by oscillating waves across the sandwave surface or by the waves at the waning stage of stormy weather. It is well known that the crest trend of such ripples can provide the first approximation of the shoreline orientation.12)

## Disconformities within the sequence

### Transgressive surface of erosion (ravinement)

**Fig. 5:**
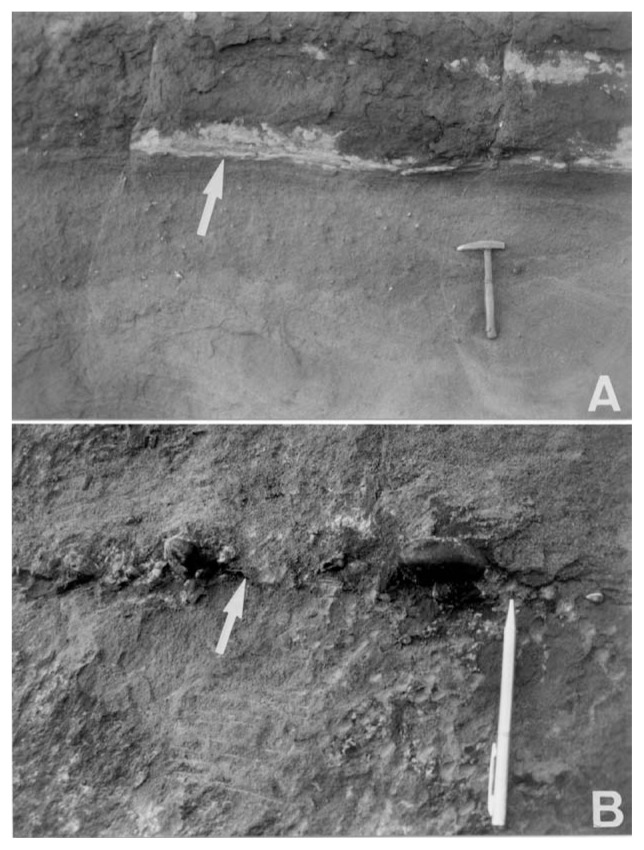
Disconformities within the sequence in Fig. 2. A. Transgressive surface of erosion (ravinement) shown by white arrow. Granulometric change and the erosive boundary are discernible. The hammer is 0.3 m long. B. Regressive surface of erosion (RSE). Pebbly sands overlie medium- to fine-grained sands. The pencil is 0.13 m long.

An abrupt facies change from facies Spb and overlying planar cross-sets to facies Fl is thought to have been caused by transgressive surface of erosion (ravinement) during transgression (Figs. 2 and 5A). The unambiguous granulometric change from medium-grained sandstone to silt or very fine-grained sandstone, together with the low-relief erosive boundary (Fig. 5A), suggests a rapid change of depositional environment from upper shoreface to middle shoreface, in which the change may have accompanied with a short-term hiatus. The reason for preserving the prism of facies Spb during transgression is discussed later.

### Regressive surface of erosion (RSE)

The explicit granulometric change from fine- to medium-grained sandstone (facies Fl) to pebbly sandstone bed shows a regressive surface of erosion (RSE) (Figs. 2 and 5B). The disparity of grain size occurs in an overall coarsening-upward succession. However, the abrupt grain size change denotes that the sea-level lowering actually took place. In contrast, such a size change never occurs through progradational sedimentation during the stable (no-lowering) sea-level.13)

## Discussion and conclusion

The bipolar or bimodal cross-stratification of facies Spb reflects the transport current reversals in the tidal inlet.14),15) However, the azimuths of such cross-stratification in the modern inlets generally show sub-parallelism to the strandline.14) The maximum dip direction of approximately E-W in facies Spb, therefore, suggests that the paleostrandline during deposition of the Taneichi Formation might have made a high angle with the present Sanriku coastline of NNW-SSE direction.

The uppermost part of the section in Fig. 1 is characterized by facies Sl, and the gently inclined internal cross-bedding shows almost E-W strike and northward dipping (Fig. 4). The average trend of ripple marks of facies Sr is N75 °W, showing a sub-parallelism to the strike direction of cross-bedding of facies Sl. As the internal cross-bedding planes generally dip seaward in the direction of progradation11) and the wave ripples produced on the surface of sand waves usually show parallel or sub-parallelism to the coastline, it is certain that the paleostrandline of the Taneichi Formation again made the high angle with the present coastline.

Erosion of nearshore sediments by scouring wave-action generally occurs during transgression, and foreshore and uppermost shoreface facies are scarcely incorporated into the transgressive stratigraphic record.16) The reason for the preservation of facies Spb in the transgressive succession, however, lies in that the sediments of bipolar cross-bedding in the tidal inlet were very thick, and bevelling by wave-action was unable to strip off all of the inlet sediments.17) The thick channel-fill sediments in the inlet throat are one of the features in the tide-dominated lagoon system.18) Other outcrops (Herukei and Yagi in Fig. 1) also show the preferential preservation of facies Spb of bipolar crosss-tratification in the transgressive successions, suggesting the presence of several tide-dominated lagoons along the Late Cretaceous coast.

About 50 km south of the studied area the Kunitan Formation (mostly Santonian but the uppermost is Campanian)19) shows the thick fluvial (regressive) and marine (transgressive) sediments filled up in an incised valley system.20) Sediments from ravinement to RSE of the studied succession are of about only 30 m thickness (Fig. 2). Compared with the thickness of the Kunitan Formation (about 60 m thick) for the same sea-level fluctuation interval (i.e., from ravinement to RSE), shelf sediment cover in this study is fairly thin. The relatively thin sediments were formed by a small amount of sediment influx from the broad but low relief landmass, as evidenced by the existence of facies Spb at several outcrops.
